# O074: EPIPA, a point prevalence survey of urinary, pulmonary and skin infections in 334 French nursing homes

**DOI:** 10.1186/2047-2994-2-S1-O74

**Published:** 2013-06-20

**Authors:** A Vincent, O Baud, N Armand, G Gavazzi, A Savey, P Fascia

**Affiliations:** 1 ARLIN Rhône-Alpes, Saint Genis Laval, France; 2 ARLIN Auvergne, Clermont-Ferrrand, France; 3 Valence General Hospital, Valence, France; 4 University Hospital of Grenoble, Grenoble, France; 5 CCLIN Sud-Est, Saint Genis Laval, France

## Introduction

Since 2009, nursing homes (NHs) in France have developed their own infection prevention and control programs. Surveillance could help these facilities put in place efficient preventive measures at a local scale.

## Objectives

To provide the participating NHs with baseline data in order to plan future prevention programs.

## Methods

A point-prevalence survey of urinary tract infections (UTI), respiratory tract infections (RTI) and skin and soft tissues infections (SSTI), as well as a survey about organisation and policies, were undertaken in the NHs of 6 regions in the South-East of France. As a validation study, 10% of the forms collected in a designated region (Rhône-Alpes) were controlled by external healthcare workers (infection control practitioners or nurses).

## Results

A total of 334 NHs (28,345 residents) were included in this survey: 80.4% of the NHs (267 out of 332) are developing an infection control program. 58.1% (193/332) of the facilities have an infection control practitioner or infection control nurse (ICT). The proportion of healthcare professionals who have received a specific formation on the prevention of Hospital Acquired Infections (HCAI) was considerably higher in NHs disposing of a part-time ICT than in those without ICT, respectively 72.7% (136/187) and 27.3% (51/187), p<0.001. Among the 28 345 residents who were questioned, 1 262 (4.45%) had at least one UTI, RTI or SSTI. SSTI are the most frequent infection with an overall prevalence of 2.08% (591/28345), followed by RTI - 1.56% (442/28345) - and finally UTI - 1.33% (378/28345). The antimicrobial prevalence was of 3.86% - 1095/28345. Answers to surveys by external ICT yielded 85.6% (95/111) of validations.

## Conclusion

This is the first cross-sectional point prevalence study of this importance in France. It was undertaken by the NHs themselves, and the collected data’s quality proves that this methodology is reliable. An impact study is programmed after this first prevalence study in order to assess practical measures which may have been developed and implemented in the NHs based on their prevalence results.

## Disclosure of interest

None declared.

